# Supplementary material for: Content-rich biological network constructed by mining PubMed abstracts
Source: BMC Bioinformatics. 2004 Oct 8;5:147. doi: 10.1186/1471-2105-5-147 (PMC528731; doi:10.1186/1471-2105-5-147)
Supplement: Additional File 2 — The original results of the above study (non-essential files are deleted to keep the file size under the limit set by BMC bioinformatics). [file 1471-2105-5-147-S2.bz2 › chilibotAdditionalFile2/dip10/753ID9852089E18138/html/DNAA_DNAB.html]

 


 **DNAA** and **DNAB** 
  
Found 99 abstracts in PubMed, retrieved 10.  
 

 What does Google say? 
 PDF only 
| .edu only 

---

**Interactive relationship** (e.g. stimulation, inhibition, etc)

**Neutral relationship**- Escherichia coli  **DnaA**  protein loads a single  **DnaB**  helicase at a  **DnaA**  box hairpin.  Ref: 12161435 J Biol Chem, 2002
- Other evidence suggests that proline 28 of  **DnaA**  is involved in the recruitment of  **DnaB**  to oriC.  Ref: 12864864 Mol Microbiol, 2003
- That only two  **DnaB**  hexamers are bound, one for each replication fork, suggests that the binding of additional molecules of  **DnaA**  in forming the prepriming complex restricts the loading of additional  **DnaB**  hexamers that can bind at oriC.  Ref: 11551962 J Biol Chem, 2001
- The  **DnaA**  replication initiation protein has been shown to be essential for DNA strand opening at the AT rich region of the replication origin of the Escherichia coli chromosome as well as serving to recruit and position the  **DnaB**  replicative helicase at this open region.  Ref: 12835421 Proc Natl Acad Sci U S A< MedlineTA>Proc Natl Acad Sci U S A, 2001

**Non-interactive relationship** (e.g. studied together, co-existance, homology, etc.)

- Thus, the TrfA 44 initiation protein has the multifunctional properties of recruiting and positioning an active form of the  **DnaB**  helicase at the RK2 replication origin by a  **DnaA**  independent process.  Ref: 12835421 Proc Natl Acad Sci U S A< MedlineTA>Proc Natl Acad Sci U S A, 2001
- Stoichiometry of  **DnaA**  and  **DnaB**  protein in initiation at the Escherichia coli chromosomal origin.  Ref: 11551962 J Biol Chem, 2001
- This is based on the competition between the lambda P protein and the host  **DnaA**  and DnaC proteins for  **DnaB**  helicase.  Ref: 11429468 Microbiology, 2001
- The AT rich region could be unwound by E. coli  **DnaA**  protein, and the  **DnaB**  helicase was loaded into the single stranded bubble.  Ref: 11606418 EMBO Rep, 2001
- In the prepriming complex, the stoichiometries are 10  **DnaA**  monomers and 2  **DnaB**  hexamers per oriC plasmid.  Ref: 11551962 J Biol Chem, 2001
- Since  **DnaA**  induces a local unwinding of oriC, one model is that the limited availability of single stranded DNA at oriC restricts the number of  **DnaB**  molecules that can bind.  Ref: 12161435 J Biol Chem, 2002
- Strand specific loading of  **DnaB**  helicase by  **DnaA**  to a substrate mimicking unwound oriC.  Ref: 12421318 Mol Microbiol, 2002
- The Escherichia coli PriA protein loads the  **DnaB**  replicative helicase at branched DNA structures independently of the replication initiator protein,  **DnaA** , and thereby facilitates assembly of functional replisomes at sites removed from oriC.  Ref: 12581361 Mol Microbiol, 2003
- However, as shown in this study, TrfA 44 specifically interacts with the  **DnaB**  replicative helicase of Pseudomonas Pseudomonas putida and Pseudomonas aeruginosa and initiates the formation of a prepriming open complex in the absence of  **DnaA**  protein.  Ref: 12835421 Proc Natl Acad Sci U S A< MedlineTA>Proc Natl Acad Sci U S A, 2003
- In E.coli DnaT and DnaC are responsible for loading  **DnaB**  onto the DNA during primosome assembly, in the PriA and  **DnaA**  mediated cascades, respectively.  Ref: 11842108 Nucleic Acids Res, 2002
